# Determining the burden of secondhand smoke exposure on the respiratory health of Thai children

**DOI:** 10.1186/1617-9625-11-7

**Published:** 2013-03-18

**Authors:** Naowarut Charoenca, Nipapun Kungskulniti, Mathuros Tipayamongkholgul, Dusit Sujirarat, Sorasak Lohchindarat, Jeremiah Mock, Stephen Lorin Hamann

**Affiliations:** 1 Faculty of Public Health, Mahidol University, Bangkok, Thailand; 2 Center of Excellence on Environmental Health and Toxicology, Bangkok, Thailand; 3 Queen Sirikit National Institute of Child Health, Bangkok, Thailand; 4 Center for the Study of Communication-Design, Osaka, Japan; 5 Tobacco Control Research and Knowledge Management Center, Bangkok, Thailand

**Keywords:** Thailand, Secondhand smoke, Children under five, Socioeconomic status, Acute lower respiratory conditions

## Abstract

**Background:**

The impact of secondhand smoke (SHS) on Southeast Asian children’s health has been assessed by a limited number of studies. The purpose of this study was to determine whether in Thailand, pre- and postnatal exposure to SHS is associated with acute lower respiratory conditions in young children.

**Methods:**

We conducted a case control study of 462 children under age five admitted with acute lower respiratory illnesses, including asthma and pneumonia, at a major hospital in Bangkok. We selected 462 comparison controls from the well-child clinic at the hospital and matched them by sex and age. We used a structured questionnaire to collect information about exposure to SHS and other factors. We conducted bivariate and multivariate analyses to identify risk factors for acute lower respiratory conditions.

**Results:**

The number of cigarettes smoked at home per day by household members was significantly greater among cases. A greater number of household caregivers of cases held and carried children while smoking as compared to controls (26% versus 7%, p <0.05). Cases were more likely to have been exposed to SHS in the household (adjusted OR = 3.82, 95% CI = 2.47-5.9), and outside (adjusted OR = 2.99, 95% CI = 1.45-6.15). Parental lower educational level and low household income were also associated with respiratory illnesses in Thai children under five.

**Conclusions:**

Thai children who are exposed to SHS are at nearly 4 times greater risk of developing acute lower respiratory conditions. Continued effort is needed in Thailand to eliminate children’s exposure to SHS, especially at home.

## Introduction

Studies conducted in high-income countries have established that secondhand smoke (SHS) exposure is harmful to respiratory health of both adults and children [1,2]. A working group at the International Agency for Research on Cancer (IARC) reviewed 9 studies on SHS exposure and concluded that SHS causes sudden infant death syndrome (SIDS), acute and chronic middle ear disease, and acute lower respiratory illness [3]. In addition, the working group found that SHS is a risk factor for asthma and increases in the severity of asthma episodes and symptoms, and impairs lung growth. To date, research on the consequences of SHS exposure among children in Thailand has been limited, and much more needs to be done to highlight this inadequately investigated threat to children in countries where the smoking prevalence is still very high [4]. Studies in Thailand have looked at SHS related to particular types of respiratory infection, fetal exposure as measured by cotinine in meconium in a small number of births, and through survey results on smoking prevalence and other factors related to SHS exposure [5-7]. Countries with available resources have been able to do larger, more extensive studies of SHS exposure and its association with respiratory and other conditions in children under five years of age [8,9]. Research has established that SHS increases childhood morbidity and mortality from respiratory infections. Yet, understanding that SHS adversely affects children’s respiratory health does not provide detail about specific factors that may lead to exposure and result in acute lower respiratory conditions [10-12].

We conducted this study to determine whether in Thailand pre- and postnatal exposure to SHS is associated with acute lower respiratory conditions in young children. We also wanted to determine whether socioeconomic status might play a role in exposure and respiratory conditions.

## Methods

We used a case–control design with sample selection by outcome to determine whether children with acute respiratory conditions (cases) had a higher probability of having been exposed to SHS than healthy children (controls).

The human subject protection protocol in this study was approved by the Research Ethics Committee of Queen Sirikit National Institute of Child Health. To recruit participants, we contacted the parents or responsible caregivers of the children we wished to include in the study, informed the parents or responsible caregivers about the study, and then we asked them for their voluntary signed informed consent.

For cases, we selected children ages 1–59 months who were admitted as inpatients in the Queen Sirikit National Institute of Child Health hospital in Bangkok who met our inclusion criteria of having a diagnosis of a SHS-sensitive respiratory condition. We included children who were diagnosed by a pediatric respiratory specialist as having an acute lower respiratory tract infection (i.e., acute laryngotracheobronchitis, acute bronchitis, acute bronchiolitis and pneumonia) or being admitted for an acute attack of bronchial asthma. We excluded children with a diagnosis of congenital heart disease, chronic lung disease, tuberculosis, nosocomial pneumonia, aspiration pneumonia, immuno-suppressive therapy, immuno-compromised host, and AIDS. Using this procedure, we obtained 462 cases. We conducted structured interviews with the child’s parent or responsible caregiver (e.g., grandparent). For controls, we selected healthy children who were seen at the Institute well-child clinic during the same time period. We matched controls to cases based on age and sex and conducted structured interviews.

### Statistical analysis

We analyzed differences between the two groups in levels of exposure to SHS using a chi-squared test. To identify potential predictors of children’s acute respiratory condition, including exposure to SHS, we conducted multiple logistic regression analysis. In our regression model, we included measures of different types of SHS exposure, and we included measures of sociodemographic status including household income and parental education to control for those differences between the two groups – cases admitted with acute lower respiratory illness and a healthy comparison group.

## Results

To determine the effectiveness of our matching procedure, we conducted an analysis of socio-demographic measures of cases compared to controls. We found that the cases and controls were well matched in terms of age and sex, but that the two groups differed significantly on several other socio-demographic measures (Results and Discussion and Table 1). In both groups, 61% were male and 70% were ages 1–24 months. In both samples, about 4/5 had a birth weight ≥2,500 grams, with a somewhat higher proportion of children who had low birth weight among the cases. Significantly more cases had been breastfeed. Somewhat more cases had parents as primary caregivers. Significantly more cases lived in poor or low-income families (66% vs. 41%). Similarly, more cases were raised in households where the father’s education level and mother’s education level were lower than among the comparison group.

**Table 1:** Comparison between sociodemographic characteristics of cases and controls

**Characteristics**	**Cases**	**Controls**	P-value*
	**n /%**	**n /%**	
**Sex**			
M	283 / 61.3	286 / 61.9	0.84
F	179 / 38.7	176 / 38.1	
**Age**			
1-12 months	182 / 39.5	192 / 41.8	0.96
12-24 months	141 / 30.6	133 / 29.0	
25-36 months	69 / 15.0	69 / 15.0	
37-48 months	44 / 9.5	41 / 8.9	
49-59 months	25 / 5.4	24 / 5.3	
**Birth weight**			
<2,500 grams	94 / 20.3	74 / 16.0	0.09
≥2,500 grams	368 / 79.7	388 / 84.0	
**Breastfeeding**			
Yes	141 / 30.5	109 / 23.6	0.01
No	312 / 67.5	350 / 75.8	
Uncertain	9 / 2.0	3 / 0.6	
**Primary caregiver**			
Mother	254 / 55.0	234 / 50.5	0.10
Father	16 / 3.5	9 / 1.9	
Others	192 / 41.5	219 / 47.4	
**Household monthly income (US $)**			
Min-max	33-5,000	56-13,333	
<150	34/ 7.4	12/ 2.6	0.01
151 - 333	272/ 59.0	174/ 37.9	
334 - 666	113/ 24.5	160/ 34.9	
≥667	42/ 9.1	113/ 24.6	
**Father’s education level**			
Grade 6 or less	189/ 41.7	102/ 22.7	0.03
Secondary or vocational school	208/ 45.9	219/48.7	
Bachelor’s degree and above	56/ 12.4	129/28.6	
**Mother’s education level**			
Grade 6 or less	192/ 41.8	120/ 26.2	0.03
Secondary or vocational school	207/ 45.1	215/ 46.8	
Bachelor’s degree and above	60/ 13.1	124/ 27.0	

*Chi-squared test, α = 0.05.

Bivariate analyses showed that during gestation, the two groups of Thai children were exposed in utero to toxins in cigarette smoke (Table 2). As indicated in Table 2, very small proportions of children in both groups were exposed during gestation directly from mother’s smoking during pregnancy, and on average those who were exposed during gestation received minimal doses from mother’s smoking. By contrast, well over two-thirds of children in both groups were exposed to SHS during gestation through maternal exposure to SHS. Significantly more cases were exposed to SHS in this way.

**Table 2:** Comparison of smoking and SHS exposure variables between cases and controls

**Measures**	**Cases**	**Controls**	**P-value**
	**n /%**	**n /%**	
Mother smoked during pregnancy*			
Yes	24 / 5.2	17 / 3.7	0. 263
No	438 / 94.8	445/ 96.3	
Average number of cigarettes mother smoked per day during pregnancy **			
Range 0–20 per day, mean ± SD	0.25 ±1.46	0.17 ±1.26	0.40
Child’s exposure to SHS during pregnancy*			
Yes	147 / 31.8	95 / 20.6	0.01
No	315 / 68.2	367 / 79.4	
Number of smokers in household*			
1-2 persons	271 / 93.1	236 / 97.1	0.036
3-4 persons	20 / 6.9	6 / 2.5	
4-5 persons	0 / 0	1 / 0.4	
Total number of cigarettes smoked in household per day *			
Range 1–40 per day, mean ± SD	12.08 ±7.71	9.95 ±6.90	<0.001
Child’s exposure to cigarette smoke while being held by a caregiver*			
Yes	121/26.2	31/6.7	0.01
No	341/73.8	431/93.3	
Child’s exposure to smoke produced from cooking*			
Yes	134 / 45.3	131 / 44.3	0.9
No	162 / 54.7	165 / 55.7	
*Chi-squared test			
** Student’s *t*-test			

A significantly greater proportion of cases lived in households with 3–4 smokers in comparison to the control group. The average number of cigarettes smoked in households of cases was 20% greater than in the comparison group’s households. A significantly greater proportion of cases were exposed to SHS while being held by primary caregivers who were smoking (26% vs 7%). Children’s exposure to smoke produced from cooking fumes was equally high in both groups.

Finally a logistic regression analysis was performed and indicated that low socioeconomic status strongly predicted the likelihood of children being admitted for acute lower respiratory conditions (From Table 3: Household income, adjusted OR for monthly < $50 = 3.99, 95%CI = 1.75-9.10, Father’s education, adjusted OR for Grade 6 or less = 2.07, 95% CI = 1.24-3.44). Thai children from very low-income families were four times more likely to be admitted. Additionally, children whose fathers had a grade 6 education or less were twice as likely to be admitted.

**Table 3:** Factors associated with the development of respiratory illness as assessed through a logistic regression analysis

**Factors**	**Cases**	**Controls**	**Crude OR**	**Adjusted OR**	P- value*
	**n /%**	**n /%**	**(95% CI)**	**(95% CI)**	
**Household monthly income (US $)**					
<150	34/ 7.4	12/ 2.6	7.62	3.99	<0.05
			(3.41-17.30)	(1.75- 9.10)	
151 - 333	272/ 59.0	174/ 37.9	4.21	2.72	<0.05
			(2.76-6.42)	(1.70-4.36)	
334 - 666	113/ 24.5	160/ 34.9	1.90	1.43	>0.05
			(1.21-2.98)	(0.89-2.28)	
≥667	42/ 9.1	113/ 24.6	1	1	
**Father’s education level**					
Grade 6 or less	189/ 41.7	102/ 22.7	4.00	2.07	<0.05
			(2.66-6.02)	(1.24-3.44)	
Secondary or vocational school	208/ 45.9	219/48.7	2.19	1.36	>0.05
			(1.49-3.21)	(0.88-2.12)	
Bachelor’s degree and above	56/ 12.4	129/28.6	1	1	
**Mother’s education level**					
Grade 6 or less	192/ 41.8	120/ 26.2	3.28	1.04	>0.05
			(2.20-4.89)	(0.62-1.75)	
Secondary or vocational school	207/ 45.1	215/ 46.8	1.99	1.05	>0.05
			(1.36-2.91)	(0.68-1.63)	
Bachelor’s degree and above	60/ 13.1	124/ 27.0	1	1	
**Child’s exposure in utero to SHS during pregnancy**					
Yes	147/ 31.8	95/ 20.6	1.80	1.16	>0.05
			(1.32-2.46)	(0.83-1.62)	
No	315/ 68.2	367/ 79.4	1	1	
**Child’s exposure to smoke while being held by a caregiver**					
Yes	121/26.2	31/6.7	4.88	3.82	<0.05
			(3.22-7.38)	(2.47-5.9)	
No	341/73.8	431/93.3	1	1	
**Child’s exposure to SHS outside their home**					
Yes	39/ 8.4	12/ 2.6	3.46	2.99	<0.05
			(1.79-6.69)	(1.45-6.15)	
No	423/ 91.6	450/ 97.4	1	1	

*P-value for adjusted OR, adjusted for household income and parental education.

## Discussion

The main finding in this study is that young Thai children are four times more likely to suffer a heavy burden of acute respiratory conditions such as acute bronchitis, pneumonia and bronchial asthma when they are raised in homes where 3 or 4 adults are smoking indoors and where family members are carrying children around while they are smoking.

After controlling for income, parent’s educational level, and SHS exposure during pregnancy, the multivariate analysis showed that Thai children who were exposed to SHS while being held by primary caregivers who were smoking were significantly more likely to be admitted for an acute lower respiratory condition, then controls. Having been held by caregivers who were smoking increased their likelihood of being admitted for an acute lower respiratory condition four-fold.

The multivariate analysis also showed that being exposed to SHS outside their homes was also a significant predictor of respiratory status. However, the proportions exposed in both groups were low (8% vs. 2%), suggesting that this mode of SHS exposure was of less importance.

Our analysis also shows that some Thai children who develop respiratory conditions are exposed to toxins in utero. This suggests that young Thai children’s exposure to SHS is a continuation of a household pattern of mothers being exposed to SHS during pregnancy. We suspect that for this reason, the multivariate model did not show this variable to be a significant predictor of respiratory status.

In the background of our analysis of the associations between SHS exposure and acute lower respiratory conditions, we found that children admitted are generally from poor or low-income households where money is tight for spending on primary care. Since the parent’s education level is also independently associated with respiratory status, we can infer that income and health awareness are both important factors influencing how children are raised, and when they receive care.

Through selecting our comparison group from a well-child clinic, we learned that parents who seek preventive primary care services for their under-fives tend to have somewhat higher incomes and be better educated. This suggests that disposable income and health awareness are important determinants of Thai children’s health beyond the scope of SHS exposure.

There are some limitations to this study. The samples were generated from one hospital and one well-child clinic in Bangkok. While it might have been preferable to select samples from multiple institutions, the available information indicates that this hospital and clinic are quite typical of such healthcare service providers. The available information also indicates that families who sought care at this hospital and clinic are like families who seek care at similar healthcare service providers throughout Bangkok and Central Thailand. Thus, we believe our findings are generalizable to much of the wider poor and low-income Thai population.

Additionally, the sample size was not as large. Nevertheless, the sample size provided adequate power for conducting robust bivariate and multivariate analyses. To generate an adequate sample size, we had to include children who were admitted with a variety of respiratory conditions rather than a single condition that is sensitive to SHS exposure. The analysis suggests that the fact of having a lower respiratory condition severe enough to require hospitalization was a sufficient criterion for measuring an outcome associated with SHS exposure.

Our original intended matching procedure did not produce samples that were sufficiently comparable to allow us to analyze the data using statistical tests appropriate for a case–control design. We therefore took a more conservative approach to analyzing the data.

We decided to rely on self-reported smoking status and self-reported exposure to SHS without obtaining biochemical verification. Self-reports can be subject to social desirability bias that can produce underestimates of smoking or overestimates of SHS exposure [13]. The data produced in this study may be subject to this bias, however the self-reported data are consistent with findings in other studies on Thai women’s smoking and SHS exposure levels [14]. While biochemical verification might have been useful for verifying parents’ current smoking status, this would not have produced reliable estimates of smoking during pregnancy or estimates of long-term exposure to SHS because cotinine has a half-life of about 2 days. For this reason, we felt the potential utility of biochemical verification for our analysis was outweighed by the cost.

## Conclusions

Our results show the need for action to educate parents and engage male smokers with knowledge of the serious harms to children from exposure to tobacco smoke.

Educational measures must be instituted so that all parents and caregivers understand the importance of smoke-free households for respiratory health, and this message should be conveyed along with efforts for smoke-free public places by health professionals and educators.

Individuals and families need to be educated about the dangers of SHS through many channels, including by health care professionals and community institutions that establish smoke-free goals. Community based projects which provide recognition to families who establish and pledge to maintain smoke-free homes have been launched and are ongoing in Thailand and should become more widespread to encourage families to proudly make their homes smoke-free.

## Competing interests

No competing interests are declared.

## Authors’ contributions

This study was conceptualized, designed, and implemented by NC, NK, SL, JM and SLH. Epidemiological and statistical contributions were made by MT and DS. All researchers contributed to the writing and review of the manuscript. All authors read and approved the final manuscript.
